# An Unusual Presentation of the Cervicobrachial Variant of Guillain-Barré Syndrome in a 32-Year-Old Previously Healthy Male

**DOI:** 10.7759/cureus.53610

**Published:** 2024-02-05

**Authors:** Samar Iltaf-Mairajuddin, Raheel Muneer Ahmed Channa, Abubaker Abdul Rahman Shaffi Al Madani, Jihad Said Inshasi, Jai Perkash, Syed Habib Ullah Kamran

**Affiliations:** 1 Neurology, Rashid Hospital, Dubai, ARE

**Keywords:** cervicobrachial (cb), neurological disorders, ivig therapy, upper limb weakness, guillain-barré syndrome

## Abstract

Guillain-Barré syndrome (GBS) is a rare autoimmune disorder characterized by acute peripheral nerve demyelination. The cervicobrachial (CB) variant presents with predominant upper limb weakness and has distinct clinical features. This case report aims to detail the clinical manifestations, diagnostic methodology, treatment outcomes, and broader implications of the CB variant of GBS. This case report presents a 32-year-old male, with a rare CB type of GBS, characterized by upper limb weakness and distinctive clinical features. Following a recent flu-like illness, the patient exhibited sudden onset weakness and neck pain. Neurological examination revealed proximal muscle weakness in the upper limbs with associated impaired pinprick sensation. Relevant laboratory investigations and imaging supported the diagnosis. The patient was diagnosed based on clinical suspicion, presentation, and cerebrospinal fluid (CSF) albuminocytological dissociation. The patient responded to intravenous immunoglobulin (IVIG) therapy, highlighting the importance of early recognition and intervention. The diagnostic approach involved nerve conduction studies (NCS), CSF analysis, and imaging, with normal findings on CT, MRI brain & cervical spine, and NCS. IVIG therapy resulted in significant improvement in muscle power. In conclusion, this case shows the significance of early recognition and intervention in the CB variant of GBS. The diagnostic methodology, encompassing advanced modalities, played a crucial role in confirming the diagnosis.

## Introduction

Guillain-Barré syndrome (GBS) represents a heterogeneous group of immune-mediated neuropathies characterized by acute onset and progressive symmetrical weakness, often following an antecedent infection [1]. Among the diverse clinical presentations within the GBS spectrum, the cervicobrachial (CB) variant emerges as a distinctive subtype, marked by the predominant involvement of the cervical and upper limb regions [2]. Recent advancements in the understanding of GBS, particularly this variant, have shed light on its unique clinical features and neuroanatomical considerations. The recent years have witnessed significant contributions to the literature, providing valuable insights into the epidemiology, pathophysiology, diagnosis, and management of this variant. While classical GBS predominantly manifests as a symmetrical limb weakness with or without cranial nerve involvement, the cervical and brachial type deviates itself due to its focal and asymmetric presentation [3]. Recent epidemiological studies have indicated that this variant may constitute a notable proportion of GBS cases, prompting a reevaluation of diagnostic criteria and recognition in clinical practice [4].

The distinct involvement of the cervical and upper limb regions in the CB variant poses intriguing questions regarding the underlying neuroanatomical and pathophysiological mechanisms [5]. Investigations spanning the years have explored the role of anti-ganglioside antibodies, molecular mimicry, and the inflammatory cascade in driving the unique clinical expression of this GBS subtype.

Diagnosing the atypical variant presents a clinical challenge due to its atypical presentation, often resulting in delayed recognition. Advances in diagnostic modalities during the specified timeframe have focused on refining nerve conduction studies (NCS), cerebrospinal fluid (CSF) analysis, and neuroimaging techniques to enhance accuracy and expedite diagnosis [6].

Early commencement of immune modulatory treatments is critical for optimal management. Recent studies have delved into treatment response patterns, emphasizing the prognostic significance of timely interventions and exploring personalized approaches to enhance patient outcomes [7].

Despite recent strides in understanding the CB variant, several knowledge gaps persist. Collaborative efforts across the international scientific community have been pivotal in standardizing diagnostic criteria and treatment guidelines. Research endeavors during the mentioned years have paved the way for ongoing investigations, seeking to unravel the genetic, immunological, and environmental factors contributing to the distinct clinical profile of this GBS subtype.

## Case presentation

A 32-year-old young male patient with no known concomitant illnesses originally complained of steadily worsening numbness and weakness in his upper limbs, which persisted for a single day. He was seen in the emergency room of Rashid Hospital's neurology department. The symptoms started in the morning and got worse as the day went on. The patient was unable to grip objects or lift his arms above his head due to a weakness that was more noticeable proximally than distally. There was no weakness or numbness in the lower limbs. No history of neck pain, trauma, joint pain, rashes, mouth ulcers, headache, vomiting, impaired vision, vertigo, or dizziness. He had a fever and flu two weeks before developing these symptoms.

After informed consent, thorough clinical evaluations were conducted, including detailed medical history, neurological examinations, and relevant laboratory investigations. The examination focused on the pattern and progression of weakness, sensory disturbances, and reflex abnormalities, particularly emphasizing the upper limb and cervical regions.

Upon examination, the patient was awake, alert, and oriented to person, place, and time. His speech was normal. Cranial nerves were all normal.

The motor exam showed normal muscle bulk throughout, no fasciculations were present, and no abnormal involuntary movements were noted. Power was 2/5 as per the Medical Research Council scale, in both upper limbs, proximally weaker than distally, and hand grip was 3/5 with absent deep tendon reflexes bilaterally at the biceps, triceps, and brachioradialis muscle. While there was normal power, tone, and reflexes in both lower limbs. Coordination, sensory examination, and gait were unremarkable.

A lumbar puncture was performed on the first day of the presentation to examine the CSF for increased protein levels, cell counts, and cultures. The presence of albuminocytological dissociation, as shown in Table 1, helped to establish the diagnosis of GBS.

**Table 1:** CSF Microscopy and Culture CSF albuminocytological dissociation (protein high and normal cell count) CSF: Cerebrospinal fluid

CSF total protein and glucose & CSF Culture-Anaerobic and Aerobic and Gram Stain/Cell Count	
CSF, Protein	
Range: 15 - 45 mg/dL	50 High
CSF, Glucose	
Range: 40 - 76 mg/dL	110 High
Macro And Micro	Appearance: pale yellow clear ((reference range-clear colorless)).
Macro And Micro	Blood: not visible coagulum: not visible
Macro And Micro	WBC count/CMM: 02 ((reference range-0-5))
Macro And Micro	RBC COUNT/CMM: 05 ((reference range-Not seen)).
Macro And Micro	Gram stain: no organisms seen ((reference range-No organism seen)).
Macro And Micro	Clear colorless supernatant with red deposit nurse notified
Culture	No growth

**Figure 1:**
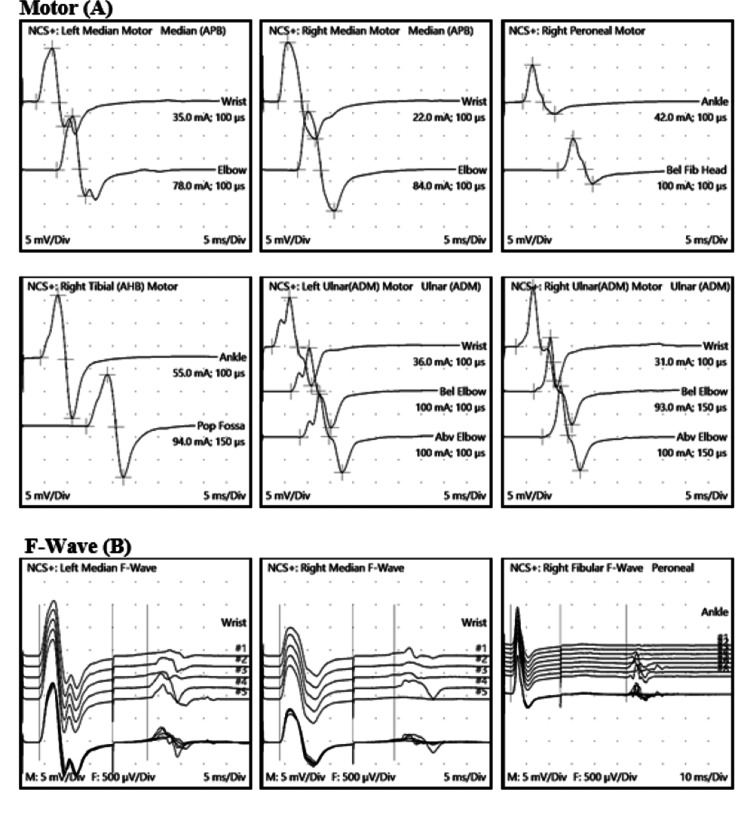
Nerve Conduction Studies: (A) Motor Conduction Velocities and (B) F wave Bilateral ulnar, median, right tibial, and right peroneal nerves showed normal motor conduction latencies, amplitudes, and conduction velocities with normal F wave latencies.

**Figure 2:**
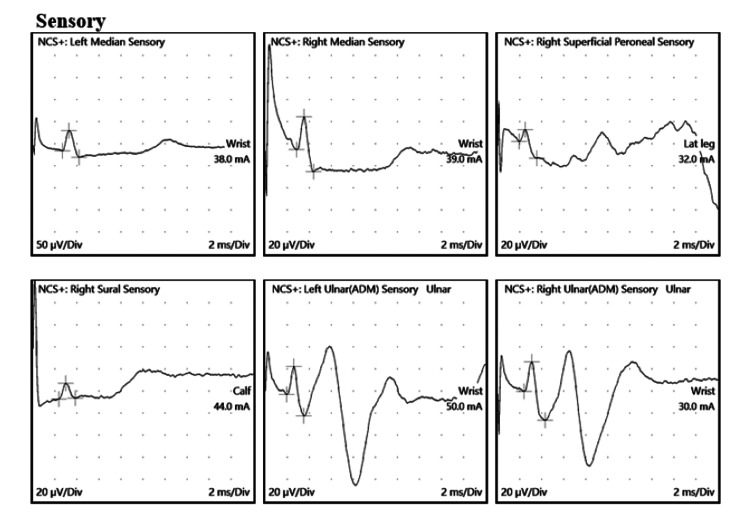
NCS: Sensory Conduction Studies Bilateral median, ulnar, right tibial, right peroneal nerves, and sensory conduction studies showed normal latencies, amplitudes, and conduction velocities. NCS: Nerve conduction studies

NCS were carried out on the fifth day, with an emphasis on upper limb nerves, and results suggestive of normal motor and sensory conduction velocities, latencies, and amplitudes were observed with normal F wave latencies during standard NCS (Table 2 and Figures 1, 2).

**Table 2:** Nerve Conduction studies (Motor, Sensory and F Wave Latencies, Amplitudes, and Conduction Velocities) Nerve conduction studies are within normal limits, with no electrophysiological evidence of large fiber polyneuropathy. No conduction block or temporal dispersion is noted in the study.

Motor Sites							
	Onset Lat	Peak-Peak Amp	Neg. Amp	Onset Lat Diff	Section Header	Distance	CV
Site	ms	mV	mV	ms	Segment	mm	m/s
Left Median (APB) Motor							
Wrist	3.2	17.2	11.8				
Elbow	7.7	17.6	11.9	4.5	Elbow-Wrist	250	56
Right Median (APB) Motor							
Wrist	3.6	21.2	12.9				
Elbow	7.8	21.9	12.7	4.2	Elbow-Wrist	240	57
Right Peroneal Motor							
Ankle	4.2	10.8	8				
Bel Fib Head	11.9	10	7	7.7	Bel Fib Head-Ankle	350	45
Right Tibial (AHB) Motor							
Ankle	4.1	27.1	13.8				
Pop Fossa	14.1	22.6	11.4	10	Pop Fossa-Ankle	420	42
Left Ulnar (ADM) Motor							
Wrist	2.2	19.5	10.9				
Bel Elbow	6.4	17.6	9.5	4.2	Bel Elbow-Wrist	215	51
Abv Elbow	8.3	17.3	9.6	1.9	Abv Elbow-Bel Elbow	100	53
Right Ulnar (ADM) Motor							
Wrist	2.7	20.7	13.2				
Bel Elbow	6.6	19.2	11.9	3.9	Bel Elbow-Wrist	225	58
Abv Elbow	8.5	18.6	11.3	1.9	Abv Elbow-Bel Elbow	105	55
F-Wave Sites							
	M-Lat	Min F-Lat					
Site	ms	(ms)					
Left Median F-Wave							
Wrist	3.9	27.6					
Right Median F-Wave							
Wrist	3.9	29.1					
Right Peroneal F-Wave							
Ankle	3.1	53.9					
Right Tibial F-Wave							
Ankle	3.7	54.7					
Left Ulnar F-Wave							
Wrist	2.6	28.9					
Right Ulnar F-Wave							
Wrist	3.1	29.5					
Sensory Sites							
	Start Lat	Lantency (Peak)	Amplitude (P-P)	Neg Amp	Segment	Distance	Start CV
Site	ms	ms	µV	µV		mm	m/s
Left Median Sensory							
Wrist-Dig II	2.7	3.3	61	46	Wrist-Dig II	145	54
Right Median Sensory							
Wrist-Dig II	2.9	3.5	50	30	Wrist-Dig II	160	55
Right Superficial Peroneal Sensory							
Lat leg-Ankle	1.95	2.5	26	11	Lat leg-Ankle	100	51
Right Sural Sensory							
Calf-Lat Mall	2.4	3	13	14	Calf-Lat Mall	120	50
Left Ulnar Sensory							
Wrist-Dig V	1.95	2.6	45	25	Wrist-Dig V	120	62
Right Ulnar Sensory							
Wrist-Dig V	2.4	3.1	53	27	Wrist-Dig V	130	54

**Figure 3:**
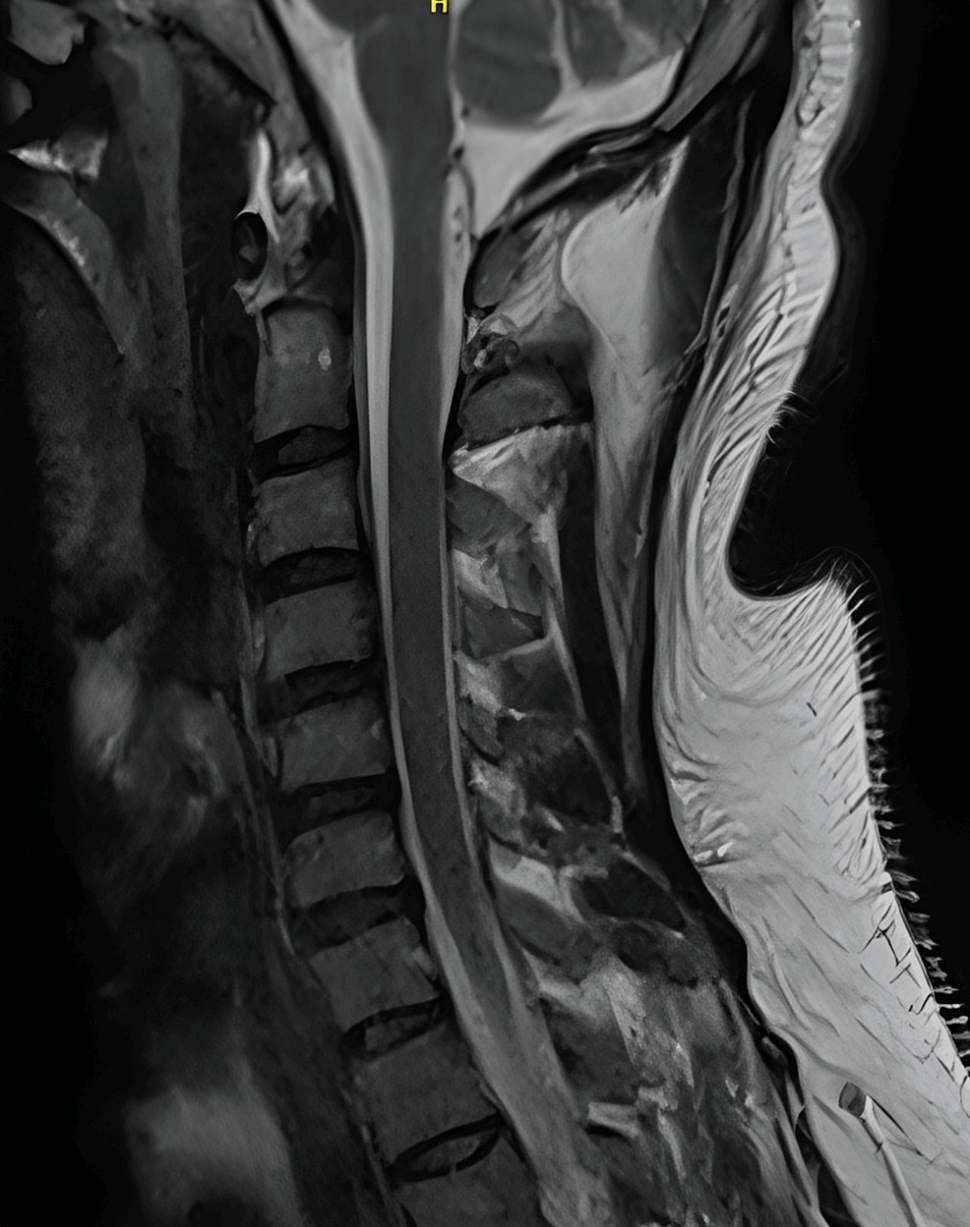
MRI Cervical Spine (T2-Weighted Sagittal Section) MRI Cervical Spine: The cervical vertebrae appear normal in height, contour, and signal intensity.  Cranio-cervical junction appears normal.  No focal bony lesion could be seen. The intervertebral discs are confined to the outlines of the corresponding endplates and vertebral bodies and maintain normal height and signal intensity. The cervical spinal cord appears normal in diameter and signal intensity.

**Figure 4:**
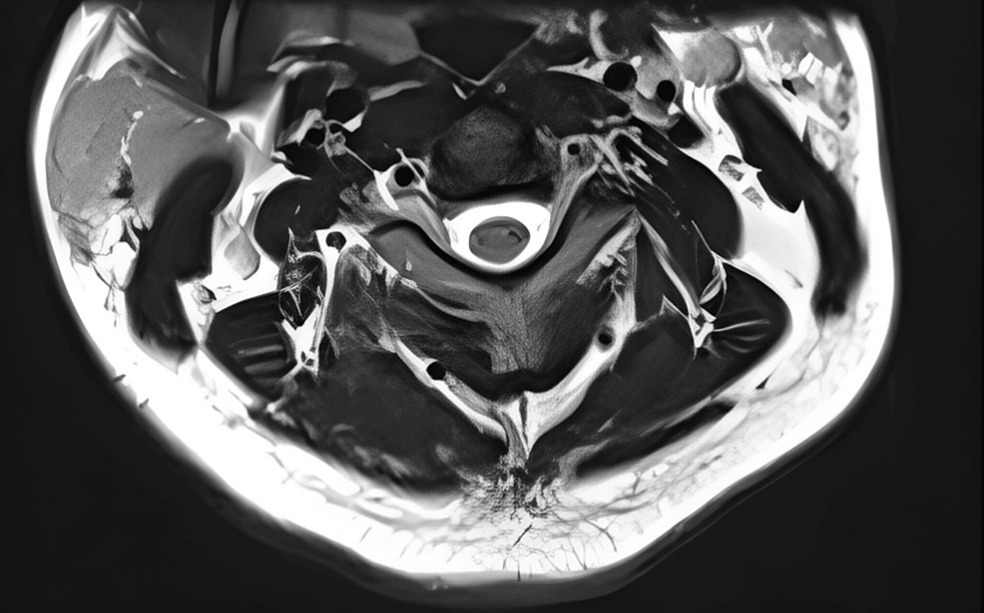
MRI Cervical Spine (T2 Axial view) MRI Cervical Spine: The cervical spinal cord appears normal in diameter and signal intensity. No focal intra or extramedullary lesion is noted.

Complementary imaging studies, such as magnetic resonance imaging (MRI) of the brain, spine, and nerve roots, were employed to rule out alternative causes and provide additional insights into the extent of cervical involvement, MRI cervical spine appears normal, with no intramedullary lesion and no root enhancement (Figures 3, 4).

Laboratory tests included a comprehensive panel to rule out infectious and autoimmune etiologies mimicking GBS. This encompassed serological tests for viral and bacterial infections, autoimmune markers, and other relevant investigations to exclude potential differentials.

Physiotherapy, close monitoring of respiratory function, and observation for any deterioration are also part of the plan. After three IVIG doses, the patient's proximal power improved to a 4/5, indicating a considerable improvement. He completed 5 doses of IVIG and was discharged with instructions to undergo an EMG NCS at three-week follow-up.

Detailed follow-up assessments were conducted to monitor the patient's response to treatment, progression of symptoms, and any potential complications. The outcomes were recorded and included a functional recovery with an mRs score of 2, with mild proximal residual weakness in both upper limbs. Despite this, the patient was able to carry out his daily tasks with ease, without assistance. The patient’s quality of life improved significantly.

This study adhered to ethical guidelines, including obtaining informed consent from the patient for the publication of the case report while ensuring confidentiality and privacy.

Given the nature of this case report, statistical analysis may not be applicable. However, if relevant, any quantitative data obtained during the diagnostic or follow-up processes should be analyzed using appropriate statistical methods.

## Discussion

The CB variant of GBS, characterized by predominant upper limb and cervical involvement, remains a distinctive clinical entity within the spectrum of GBS [8]. The variability in GBS phenotypes, including this variant, emphasizes the need for a nuanced understanding to facilitate early diagnosis and targeted therapeutic interventions. A comprehensive study identified specific clinical markers associated with this variant of GBS, emphasizing the importance of early clinical recognition. The unique involvement of the cervical region raises questions about the specific neuroanatomical pathways affected. Recent studies have elucidated the role of antiganglioside antibodies and molecular mimicry in the pathophysiology of GBS [9]. Advances in neuroimaging techniques during this period have provided a more detailed examination of the affected regions, contributing to a deeper understanding of the pathophysiology. Diagnosing the CB variant can be challenging due to its uncommon presentation. Advances in diagnostic techniques have been made during this time, with an emphasis on enhancing accuracy by combining NCS, CSF analysis, and neuroimaging [10]. High-resolution ultrasound, introduced in recent years, has shown promise in detecting early nerve involvement, aiding in the diagnostic process. Evaluating the response to standard GBS treatments, such as IVIG or plasma exchange, is crucial. Studies from the past decade have emphasized the prognostic significance of early initiation of immune-modulatory therapies and their impact on long-term outcomes [11]. Throughout this time, attempts have been made to investigate more individualized and successful treatment strategies based on unique patient features [12]. A comparative analysis between the CB variant and classical GBS presentations may reveal distinctive features in terms of clinical course, recovery trajectories, and residual deficits [13]. Genetic and immunological studies have provided insights into the differences between various GBS subtypes, contributing to a more nuanced comparison [14]. Recognition of the CB variant underscores the need for heightened clinical suspicion, especially in cases presenting with upper limb weakness. The cooperative global initiatives to create uniform treatment protocols and diagnostic standards, improve the uniformity of care across various healthcare environments [15].

Limitations of the study are that this study has a retrospective design and there is reliance on a single case report. Future research should explore ways to overcome these limitations. Collaborative efforts and larger cohort studies, especially spanning the recent decade, may provide a more comprehensive understanding of this variant.

## Conclusions

The presented case of the CB variant of GBS highlights the clinical complexity and challenges associated with this distinctive subtype. The CB variant of GBS manifests with predominant upper limb and cervical involvement, presenting a clinical challenge due to its atypical nature. Timely initiation of immune modulatory therapies, such as IVIG or plasmapheresis, plays a crucial role in improving outcomes, as evidenced by the patient's response in this case. For the comprehensive care of patients with the CB type of GBS, a prompt collaboration between neurologists, critical care physicians, and physiotherapists is crucial. To ensure consistency of care across diverse healthcare environments, standardized diagnostic criteria and treatment protocols have been made possible by collaborative efforts within the international scientific community.
